# Novel Therapeutic Target(s) for Psoriatic Disease

**DOI:** 10.3389/fmed.2022.712313

**Published:** 2022-02-21

**Authors:** Vishal Thakur, Rahul Mahajan

**Affiliations:** Department of Dermatology, Venereology, and Leprology, Postgraduate Institute of Medical Education and Research, Chandigarh, India

**Keywords:** psoriasis, psoriatic arthritis, infliximab monotherapy, autoimmune hepatitis, treatment, biologics and biosimilars, small molecule

## Abstract

Psoriasis and psoriatic arthritis, together known as psoriatic disease, is highly prevalent chronic relapsing inflammatory disease affecting skin, joints or both and is associated with several comorbidities such as cardiovascular, metabolic, psychiatric, renal disease etc. The etiopathogenesis of psoriasis is complex and mainly driven by aberrant immune response owing to the genetic susceptibility and various environmental factors such as trauma, infections and drugs. Recent advances in understanding molecular and cellular pathways have identified tumor necrosis factor-α (TNF-α), interleukin-17 (IL-17), IL-23, IL-22 as major contributors in psoriasis pathogenesis. Advances in the knowledge of pathophysiology, the interaction of autoinflammation and clinical phenotypes have led to the development of highly effective targeted therapeutic agents which include TNF-α, IL-17, IL-23, IL-1 α/β or IL-36 inhibitors or receptor blockers, small molecule drugs like phosphodiesterase-4 inhibitors (apremilast), Janus kinase (JAK) inhibitors, retinoic acid receptor-related orphan receptor γt (RORγt) inhibitors. These novel drugs have promised the potential of improved disease control. In recent years, the transition from biologics to biosimilars especially with TNF-α inhibitors had significant impact on decreasing health care cost and increasing therapeutic options to the patients. However, selection of right treatment for an individual patient still remains challenging. Moreover, interplay between different epigenetic mechanisms such as the DNA methylation, chromatin modifications and noncoding RNA regulation has recently been started to be deciphered. Enzymes inhibitors involved in epigenetic pathways such as DNA methyltransferases and histone deacetylases demonstrated to restore normal epigenetic patterns in clinical settings and have provided the potential as novel therapeutic targets for psoriasis. In this review, we will discuss novel biologic agents and newer therapeutic approaches in treatment of psoriatic disease.

## Introduction

**Figure 1:**
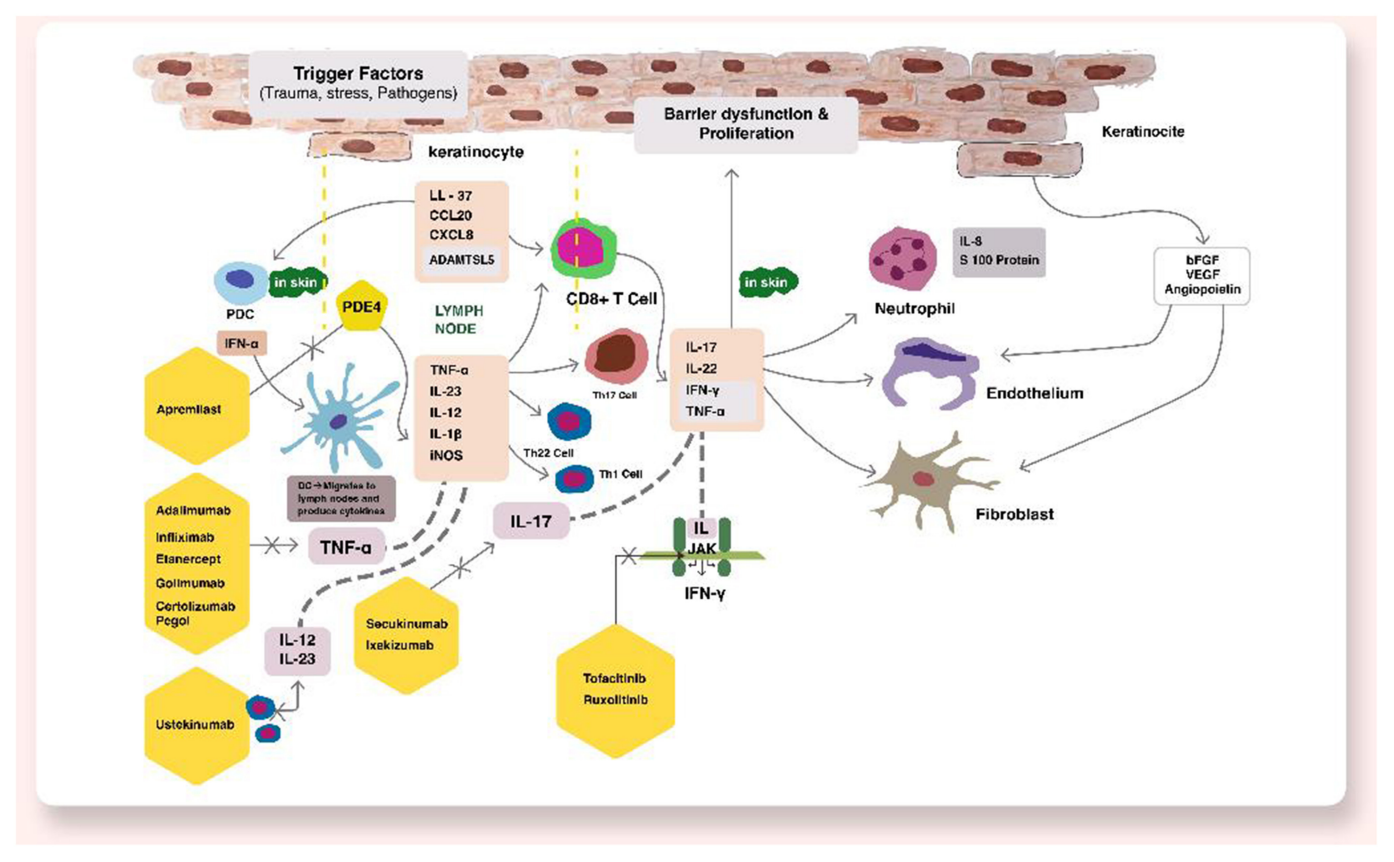
Pathogenesis and various therapeutic targets in psoriatic disease.

Psoriatic disease is a chronic relapsing inflammatory condition affecting ~2–3% of population (1, 2). Psoriatic disease consists of psoriasis vulgaris affecting skin and psoriatic arthritis affecting joints. Psoriasis affects patients' quality of life significantly and have tremendous psychosocial burden among patients (3). The immunopathogenesis of psoriasis is complex primarily driven by an aberrant immune response further modified by an interplay between genetic susceptibility and environmental factors. The inflammatory events lead to systemic inflammation resulting in cardiovascular, metabolic and renal disease and increased morbidity (4). In last few years, advances in understanding molecular and cellular pathways have identified tumor necrosis factor-α (TNF-α), interleukin-17 (IL-17), IL-23, IL-22 as major contributors in psoriasis pathogenesis (5). This has led to the development of highly effective targeted therapeutic agents which include TNF-α, IL-17, IL-23, IL-1 α/β or IL-36 inhibitors or receptor blockers, small molecule drugs like phosphodiesterase-4 inhibitors (apremilast), Janus kinase (JAK) inhibitors, retinoic acid receptor-related orphan receptor-γT (RORγT) inhibitors (5). Figure 1 shows the pathogenesis and various therapeutic targets in psoriatic disease. These novel drugs have promised the potential of improved disease control. In this review, we will discuss novel therapeutic targets in the management of psoriatic disease.

## Jak Inhibitors

The Janus Kinase–Signal Transducer and Activator of Transcription (JAK–STAT) pathway plays an important role in intracellular signaling in various physiological and pathological processes in inflammatory disorders including psoriasis. Cytokines implicated in psoriasis pathogenesis mainly IL-17, IL-23, TNF-α, IL-1, IL-22, IFN-α and IFN-γ are linked to JAK-STAT pathway (6, 7). Upon interaction of various cytokines with their respective receptor, activation of JAK leads to phosphorylation of STAT proteins and nuclear translocation resulting in gene expression (8). In psoriasis, increased expression and upregulation of STAT1 and STAT3 have been demonstrated in the lesional skin (9, 10). STAT1 and STAT3 are involved in the activation of dendritic cells and differentiation of Th1 and Th17 cells (9, 10). STAT3 also leads to the keratinocyte proliferation mediated through IL-19, IL-36 and IL-22 (11). IFN-γ secreted from keratinocytes leads to the migration of inflammatory cells from the lymphoid tissue to the skin (10).

Various JAK inhibitors have been used in psoriatic disease with good efficacy, of which Tofacitinib, an oral JAK1/3 inhibitor, has been extensively studied in phase II and III trials (6). In phase III studies, a significant proportion of patients achieved PASI75 at weeks 12 or 16 showing greater efficacy with higher doses i.e., 10 mg twice daily (12). Studies evaluating the efficacy after treatment withdrawal also showed higher efficacy as compared to placebo (13). In another study, 74.1 and 79.4% of patients receiving tofacitinib 5 mg twice daily and 10 mg twice daily respectively, maintained the response at 52-weeks (14). Tofacitinib has shown significantly better efficacy and safety in psoriatic arthritis as compared to placebo (15, 16). A topical formulation of tofacitinib has also been developed and used in plaque psoriasis with modest efficacy (17). Common adverse effects include cytopenia and infections (6, 18). Safety concerns especially dose-dependent (i.e., 10 mg twice daily) risk of herpes zoster, higher chances of infections, gastric perforation and thromboembolic events has been raised (6, 18), although long-term studies with larger samples are needed. Due to these safety concerns, tofacitinib was not approved for psoriasis by FDA, however it is approved for use in psoriatic arthritis (6).

Baricitinib, an oral highly selective JAK1 and JAK2 inhibitor has also been studied in patients with moderate-to-severe psoriasis in Phase II trials and has shown better efficacy as compared to placebo at doses 8 mg and 10 mg (19). Adverse effects included anemia, cytopenia and increase in creatinine levels (6). Similar safety concerns have been raised with baricitinib, thus, it is approved for use in rheumatoid arthritis only.

Ruxolitinib, another JAK1 and JAK2 inhibitor, has been developed as topical cream and studies in psoriasis showed a better efficacy and safety profile compared to vehicle and Non-inferior to calcipotriol-betamethasone combination (17). Other JAK1/2 inhibitors such as itacitinib (20), abrocitinib (21), solcitinib (22) and filgotinib (23) have shown efficacy in phase II trials in psoriasis and psoriatic arthritis. Peficitinib, an oral pan-JAK inhibitor with JAK3 selectivity, showed a good efficacy in psoriasis in phase IIa trial with no major adverse events (24).

## IL-23

IL-23, a cytokine of IL-12 family, consists of two subunits: p19 (unique for IL-23) and p40 that is common with IL-12 (25). IL-23 is mainly produce by dendritic cells and macrophages (26, 27). Initially, antibodies targeting p40 subunit of IL-12 were found effective in psoriasis as these neutralized IL-23 also (27). Later on, increased expression of p19 and p40 was found in psoriatic lesions while p35 that is specific to IL-12 was normal which suggested that IL-23 not IL-12 is an important cytokine involved in the psoriasis pathogenesis (26). IL-23 binds to its heterodimeric receptor leading to the activation of Janus kinases (Jak) and further activation of STAT3 (28). IL-23 leads to the production of cytokines from Th-17 cells i.e., IL-17, a major cytokine implicated in the pathogenesis of psoriasis (28). This led to development of anti-IL23 biologics in the therapeutics of psoriatic disease. As these agents target upstream cytokine involved in the psoriasis pathogenesis, dosing interval of longer duration is an advantage as compared to the downstream cytokines such as IL-17 and TNF-α (29). Currently, ustekinumab, guselkumab, tildrakizumab, and risankizumab are FDA approved for psoriasis vulgaris and only ustekinumab and guselkumab have been approved by the FDA for psoriatic arthritis (29). IL-23 inhibitors have shown superior efficacy to conventional agents and TNF-α inhibitors. A network metanalysis found guselkumab and risankizumab more effective than tildrakizumab (30). The IL-23 inhibitors have been found to be more effective in maintaining remission as compared to other drugs even after drug discontinuation. In PHOENIX 1 trial of ustekinumab, median time to loss of PASI-50 was ~22 weeks from the last dose of drug (31). Similar results have been observed with other IL-23 inhibitors including guselkumab, tildrakizumab and risankizumab, showing sustained improvement in disease after drug discontinuation (32–34). The efficacy of IL-23 inhibitors especially ustekinumab and guselkumab in psoriatic arthritis was also found significantly high as compared to placebo (35–37). However, more studies evaluating efficacy of these agents and comparison with other drugs such as TNF-α are required. Common adverse effects of IL-23 inhibitors include upper respiratory infections, nasopharyngitis, and headache (29). Other advents events include serious infections, major adverse cardiovascular events and malignancy, however, the rates observed were comparable to seen in general population of psoriasis patients (29). A long-term data on the safety of these novel drugs is thus warranted.

## IL-36

IL-36 (member of IL-1 family) binds to its receptor and leads to the activation of NF-κB and MAPKs pathways through MyD88/IRAK complex (38). Expression of IL-36γ have been found to be significantly upregulated in the serum and skin samples of psoriasis patients (39). Furthermore, loss of function mutation in IL-36Ra gene has been found in a severe variant of generalized pustular psoriasis (GPP) (40, 41). Studies in mouse model have observed psoriasis like epidermal changes, inflammatory cell infiltrate and gene dysregulation after IL-36 administration which was not seen when Pre-treatment with an IL-36 antagonist was administered (42). This supports a direct role of IL-36 in psoriasis pathogenesis and attenuating this signaling pathway may be an effective alternative approach to the already approved small molecules such as apremilast or other biologics. Moreover, studies have shown that individuals with loss of function mutation in IL-36Ra gene have normal immune function suggesting that targeting this cytokine may not lead to adverse events associated with immune dysregulation and may have a good safety profile (43). Recently, an oral small molecule inhibitor of IL-36, A-552 was shown to inhibit IL-36γ and production of other cytokines induced by IL-36γ in human and mouse cells (44). Monoclonal antibody against IL-36R, spesolimab has shown efficacy in a Phase I study, and phase II and III studies of spesolimab in GPP are currently undergoing (45, 46). Thus, anti-IL-36 agents may have a robust potential in therapeutics of psoriasis and further research evaluating their efficacy and safety is needed. Table 1 summarizes the studies of JAK inhibitors, IL-23 and IL-36 inhibitors in psoriasis and psoriatic arthritis.

**Table 1:** Summary of various trials of JAK inhibitors, IL-23, IL-12/23 and IL-36 inhibitors.

**Drug**	**Study/year**	**Setting/Dose**	**Number of patients**	**Response**	**Adverse effects**	**Conclusion**	**Phase**
JAK inhibitors Tofacitinib	Papp et al. (87)/ 2012	Psoriasis vulgaris–Tofacitinib 2 mg twice daily vs. 5 mg twice daily vs. 15 mg twice daily vs. placebo	Tofacitinib 2 mg-49; 5 mg−49; 15 mg−49; placebo-50	At week 12, higher proportion of patients achieved PASI 75 in all tofacitinib groups: 25·0% (2 mg), 40·8% (5 mg) and 66·7% (15 mg) compared with placebo (2·0%).	Infections and infestations	Oral tofacitinib results in significant clinical improvement in patients with moderate-to-severe plaque psoriasis.	Phase 2b
	Bisonette et al. (13)/ 2015	Moderate-to-severe plaque psoriasis–tofacitinib 5 mg or 10 mg twice daily for 24 weeks. The patients achieving both PASI75 and Physician's Global Assessment of “clear” or “almost clear” received a placebo or the previous dose. At relapse (>50% reduction in the PASI improvement during initial treatment) or week 40, the patients received the initial dose.	Tofacitinib 5 mg−331; 10 mg−335	33·5% and 55·2% achieved both PASI 75 and PGA responses in tofacitinib 5 and 10 mg twice daily group, respectively.	Elevations in low-density lipoprotein–cholesterol levels	Patients who received continuous treatment maintained a response more effectively than placebo. Patients who relapsed, 60% reattained a response with tofacitinib.	Phase 3
	Bachelez et al. (88) / 2015	Moderate-to-severe plaque psoriasis–Tofacitinib 5 mg twice daily vs. 10 mg twice daily vs. Etanercept 50 mg twice weekly vs. placebo	Tofacitinib 5 mg−330; 10 mg−332; Etanercept- 336; placebo- 108	At week 12, PASI75–39·5% in tofacitinib 5 mg group, 63·6% in tofacitinib 10 mg group, 58·8% in the etanercept group, and 5·6% in the placebo group.	Serious adverse events−2% in tofacitinib 5 mg group, 2% in tofacitinib 10 mg group, 2% in etanercept group, and 2% in placebo group.	Tofacitinib 10 mg twice daily was Non-inferior to etanercept and was superior to placebo, but 5 mg twice daily did not show Non-inferiority to etanercept.	Phase 3, randomized, multicentre, placebo-controlled, 12-week, Non-inferiority trial.
	Papp et al. (12)/ 2015	Plaque psoriasis–tofacitinib 10 or 5 mg or placebo, twice daily.	Tofacitinib 5 mg−745; 10 mg−741; placebo- 373	At week 16, a greater proportion of patients achieved PGA responses with tofacitinib 5 and 10 mg twice daily vs. placebo.	Similar across groups. Twelve patients reported herpes zoster across the tofacitinib treatment groups.	Oral tofacitinib demonstrated significantly high efficacy as compared to placebo, during 16 weeks of treatment.	Phase 3
	Mease et al. (15)/ 2017	Psoriatic arthritis–tofacitinib 5-mg twice daily, 10-mg twice daily, adalimumab 40-mg once every 2 weeks, placebo with a blinded switch to 5-mg tofacitinib at 3 months, or placebo with a blinded switch to 10-mg tofacitinib at 3 months.	Tofacitinib 5 mg−107; 10 mg−104; adalimumab- 106; placebo- 52 (5 mg switch), 53 (10 mg switch).	ACR20 response rates at month 3 were 50% in 5-mg tofacitinib group and 61% in 10-mg tofacitinib group, 33% in placebo group, 52% in the adalimumab group.	The rate of adverse events was 66% in 5-mg tofacitinib group, 71% in 10-mg tofacitinib group, 72% in adalimumab group.	Efficacy of tofacitinib was superior to placebo at month 3 in patients who previously had an inadequate response to conventional synthetic DMARDs.	12-month, double-blind, active-controlled and placebo-controlled, phase 3 trial
	Gladman et al. (16)/ 2017	Psoriatic arthritis–tofacitinib 5 mg twice daily; 10 mg twice daily; placebo, with a switch to 5 mg tofacitinib twice daily at 3 months; or placebo, with a switch to 10 mg tofacitinib twice daily at 3 months.	Tofacitinib 5 mg−132; 10 mg−132; placebo- 66 (5 mg switch), 65 (10 mg switch).	ACR20 response- 50% with 5-mg tofacitinib and 47% with 10-mg dose, as compared to 24% with placebo.	4 serious infections, 3 herpes zoster infections, 1 myocardial infarction, and 1 ischemic stroke.	Tofacitinib was more effective than placebo over 3 months in reducing disease activity.	6-month randomized, placebo-controlled, double-blind, phase 3 trial
Baricitinib	Papp et al. (19)/ 2016	Moderate-to-severe psoriasis- placebo or oral baricitinib at 2, 4, 8 or 10 mg once daily for 12 weeks.	baricitinib 2 mg- 32, 4 mg- 72, 8 mg- 64, 10 mg- 69, Placebo- 34	At week 12, patients in 8-mg (43%) and 10- mg (54%) baricitinib groups achieved PASI-75 than in placebo group (17%). Statistically significant PASI-90 responses were achieved in 8-mg and 10-mg groups at 8 and 12 weeks.	treatment-emergent AE rates were 44, 50, 47, 58 and 64% for placebo and 2-, 4-, 8- and 10-mg baricitinib groups.	Treatment with baricitinib for 12 weeks achieved significant improvements in PASI-75.	Phase 2b, randomized, double-blind, placebo-controlled, dose-ranging study.
IL-12/23 inhibitors Ustekinumab	Phoenix-I (89)/ 2008	Moderate-to-severe psoriasis- Ustekinumab 45 mg or 90 mg at weeks 0, 4 and then every 12 weeks; or placebo at weeks 0 and 4, with subsequent crossover to ustekinumab at week 12.	Placebo-255; 45 mg- 255; 90 mg- 256	67·1% patients receiving ustekinumab 45 mg, 66·4% receiving ustekinumab 90 mg, and 3·1% receiving placebo achieved PASI 75 at week 12.	Adverse events occurred in 54·5% in ustekinumab and 48·2% in placebo group.	Ustekinumab seems to be efficacious for the treatment of moderate-to-severe psoriasis; dosing every 12 weeks maintains efficacy for at least a year in most patients.	Phase 3, parallel, double-blind, placebo-controlled study.
	Phoenix-II (90)/ 2008	Moderate-to-severe psoriasis- Ustekinumab 45 mg or 90 mg at weeks 0, 4 and then every 12 weeks; or placebo. Partial responders (patients achieving ≥50% but <75% improvement from baseline in PASI) were re-randomized at week 28 to continue dosing every 12 weeks or escalate to dosing every 8 weeks.	Placebo- 410; 45 mg- 409; 90 mg−411	66·7% patients receiving ustekinumab 45 mg, 75·7% receiving ustekinumab 90 mg, and 3·7% receiving placebo achieved 75% improvement in PASI at week 12. More partial responders who received ustekinumab 90 mg every 8 weeks achieved PASI 75 at week 52 than those who received the same dose every 12 weeks.	Serious adverse events were seen in 2% patients in 45 mg group, 1·2% in 90 mg group, and 2% in placebo group.	Ustekinumab every 12 weeks is effective for most patients with moderate-to-severe psoriasis. Intensification of dosing to once every 8 weeks with ustekinumab 90 mg might be necessary to elicit a full response in patients who only partially respond to the initial regimen.	Multicentre, phase 3, double-blind, placebo-controlled study.
	Griffiths et al. (91)/ 2010	Moderate-to-severe psoriasis- 45 or 90 mg of ustekinumab (at weeks 0 and 4) or high-dose etanercept (50 mg twice weekly for 12 weeks)	45 mg−209; 90 mg−347; etanercept−347	75% improvement in the PASI at week 12 in 67.5% of patients receiving 45 mg of ustekinumab and 73.8% of patients receiving 90 mg, as compared with 56.8% of those with etanercept.	One or more adverse events occurred in 66% of patients in 45 mg ustekinumab and 69.2% in 90 mg ustekinumab and in 70% in etanercept group.	Efficacy of ustekinumab 45 or 90 mg was superior to high-dose etanercept over a 12-week period.	Randomized, multicentre study.
	PSUMMIT I (35)	Active psoriatic arthritis−45 mg ustekinumab, 90 mg ustekinumab, or placebo at week 0, week 4, and every 12 weeks thereafter.	Placebo- 206; 45 mg- 205; 90 mg−204	More ustekinumab-treated [42·4%] in the 45 mg group and [49·5%] in the 90 mg group than placebo-treated [22·8%] patients achieved ACR20 at week 24.	Adverse events were similar in the ustekinumab [41·8%] and placebo groups [42·0%].	Ustekinumab significantly improved active psoriatic arthritis.	Phase 3, multicentre, double-blind, placebo-controlled trial
	PSUMMIT II (36)	Active Psoriatic Arthritis- ustekinumab 45 mg or 90 mg at week 0, week 4, q12 weeks or placebo at week 0, week 4, week 16 and crossover to ustekinumab 45 mg at week 24, week 28 and week 40.	Placebo- 104; 45 mg- 103; 90 mg- 105	More ustekinumab-treated (43.8% combined) than placebo-treated (20.2%) patients achieved ACR20 at week 24; all benefits were sustained through week 52.	No unexpected adverse events were observed.	Ustekinumab (45/90 mg q12 weeks) yielded significant and sustained improvements in Psoriatic arthritis.	phase 3, multicentre, placebo-controlled trial
IL-23 inhibitor Guselkumab	VOYAGE-I (92)	Moderate to severe plaque psoriasis- guselkumab 100 mg (weeks 0 and 4, then every 8 weeks); placebo/guselkumab (weeks 0, 4, and 12 then guselkumab at weeks 16 and 20, then every 8 weeks); or adalimumab (80 mg week 0, 40 mg week 1, then 40 mg every 2 weeks through week 47).	Placebo- 174; 100 mg−329; adalimumab−334	Guselkumab was superior to placebo at week 16 (73.3 vs. 2.9% [PASI-90]). Guselkumab was also superior to adalimumab for PASI 90 at week 16 (73.3 vs. 49.7%), week 24 (80.2 vs. 53.0%), and week 48 (76.3 vs. 47.9%).	The proportions of patients with adverse events were similar in the guselkumab and adalimumab group.	Guselkumab demonstrated superior efficacy compared with adalimumab.	phase 3, randomized, double-blind, placebo- and active comparator-controlled trial
	VOYAGE-II (93)	Moderate to severe plaque psoriasis- Similar to VOYAGE I; at week 28, guselkumab PASI90 responders were rerandomized to guselkumab or placebo with guselkumab after loss of response. Placebo → guselkumab responders and adalimumab responders received placebo, then guselkumab after loss of response.	Placebo- 248; 100 mg−496; adalimumab−248	Guselkumab was superior to adalimumab and placebo at week 16. From weeks 28 to 48, better persistence of response was observed in guselkumab maintenance vs. withdrawal groups. Of adalimumab Non-responders who switched to guselkumab, 66.1% achieved PASI 90 at week 48.	Adverse events were comparable among groups.	Guselkumab is highly effective maintenance therapy, including in adalimumab Non-responders.	phase 3, double-blind, placebo- and active comparator–controlled
	DISCOVER I (37)	Active psoriatic arthritis	Placebo- 126; 100 mg every 4 weeks- 128; 100 mg at 0 and 4 weeks, then every 8 weeks- 127	Significantly greater proportions of patients receiving guselkumab every 4-week (59·4%) and every 8-week (52·0%) vs. placebo (22·2%) achieved ACR20 at week 24.	Serious adverse events occurred in none of patients in guselkumab every 4-week, 3·1% in guselkumab every 8-week, and 4·0% in placebo group.	Guselkumab demonstrated a favorable benefit-risk profile and is an effective treatment option in patients with active psoriatic arthritis.	Phase-3, double-blind, placebo-controlled study
Tildrakizumab	reSURFACE I (94)	Moderate-to-severe chronic plaque psoriasis- Tildrakizumab at weeks 0 and 4 during part 1 and at week 16 during part 2 (weeks 12 and 16 for participants re-randomized from placebo to tildrakizumab.	Placebo- 154 100 mg- 309 200 mg-308	At week 12, 62% in 200 mg group and 64% in 100 mg group achieved PASI 75, compared with 6% in placebo group.	Nasopharyngitis.	Tildrakizumab 200 mg and 100 mg were efficacious compared with placebo.	Parallel group, double-blind, randomized controlled study
	reSURFACE II (94)	Moderate-to-severe chronic plaque psoriasis- Tildrakizumab at weeks 0 and 4 during part 1 and at week 16 during part 2 (weeks 12 and 16 for participants re-randomized from placebo to tildrakizumab; etanercept was given twice weekly in part 1 and once weekly during part 2).	Placebo- 156 100 mg- 307 200 mg-314 Etanercept−313	At week 12, 66% in 200 mg group, and 61% in 100 mg group achieved PASI 75, compared with 6% in placebo group and 48% in the etanercept group.	The incidence of severe infections, malignancies, and major adverse cardiovascular events were low and similar across treatment groups.	Tildrakizumab 200 mg and 100 mg were efficacious compared with placebo and etanercept and were well tolerated.	Parallel group, double-blind, randomized controlled study
Risankizumab	UltIMMa-1 and UltIMMa-2 (95)	Moderate-to-severe chronic plaque psoriasis−150 mg risankizumab, 45 mg or 90 mg ustekinumab or placebo. Following 16-week double-blind treatment period (part A), patients initially assigned to placebo switched to 150 mg risankizumab at week 16; other patients continued their originally randomized treatment (part B, double-blind, weeks 16–52). Study drug was administered subcutaneously at weeks 0 and 4 during part A and at weeks 16, 28, and 40 during part B.	UltIMMa-1 - Placebo- 102; 150 mg−304; ustekinumab−100 UltIMMa-2- Placebo-98; 150 mg−294; Ustekinumab- 99	At week 16 of UltIMMa-1, PASI 90 was achieved by 75·3% patients receiving risankizumab vs. 4·9% receiving placebo and 42·0% receiving ustekinumab. At week 16 of UltIMMa-2, PASI 90 was achieved by 74·8% patients receiving risankizumab vs. 2·0% receiving placebo and 47·5%.	The frequency of treatment-emergent adverse events in UltIMMa-1 and UltIMMa-2 was similar across risankizumab, placebo, ustekinumab, and placebo to risankizumab groups.	Risankizumab showed superior efficacy to both placebo and ustekinumab.	Phase 3, randomized, double-blind, placebo-controlled and active comparator-controlled trials
IL-36 inhibitor Spesolimab	Bachelez et al. (96)	Generalized Pustular Psoriasis- single 900-mg intravenous dose of spesolimab or placebo. Patients in both groups received an open-label dose of spesolimab on day 8, an open-label dose of spesolimab as a rescue medication after day 8, or both and were followed to week 12.	Spesolimab 900 mg- 35; placebo- 18	At week 1, 54% in the spesolimab group had a pustulation sub-score of 0, as compared with 6% in the placebo group.	Drug reactions−2 patients. (drug-induced hepatic injury- 1); infections−17% through the first week; antidrug antibodies−46%.	Spesolimab resulted in a higher incidence of lesion clearance at 1 week than placebo but was associated with infections and systemic drug reactions.	Phase 2 randomized trial

## IL-1

IL-1, a proinflammatory cytokine, comprise of IL-1α and IL-1β. Both these cytokines has been implicated in the pathogenesis of psoriasis (46). Increased expression of IL-1β has been found in the psoriatic skin and correlated with disease severity (47). Furthermore, IL-1β has been shown to induce Th17 cells and stimulate keratinocytes to secrete chemokines such as CCL20 (47). IL-1β production is also regulated by NLRP3 inflammasome as these inflammasomes cleave procaspases into caspases leading to the production of IL-1β (48). Higher caspase-1 and IL-1β levels has been observed in patients with psoriasis that normalized after treatment with TNF-α (48). Anti-IL1 agents such as anakinra, canakinumab and gevokizumab have shown efficacy in psoriasis. Anakinra, a recombinant IL-1 receptor antagonist (IL-1Ra) inhibits both IL-1α and IL-β and has shown efficacy in pustular psoriasis and deficiency of IL-1 receptor antagonist (DIRA) variant (49). However, the partial responses observed suggest role of other cytokines of IL-1 family such as IL-36 (49, 50). Canakinumab, an anti-IL-β antibody has also shown beneficial effects in GPP (51). Gevokizumab, another novel IL-1β antagonist has shown its efficacy in GPP (52). In 2 patients of GPP, 79 and 65% improvement in GPP scores was observed after 4 weeks (52). Thus, IL-1 inhibitors particularly IL-1β could be potentially efficacious in management of psoriasis especially pustular psoriasis, though larger studies are needed.

## RorγT Antagonists

RORγT is an important transcription factor required for the differentiation of Th17 cells and regulates the expression of Th17 cytokines i.e., IL-17A, IL-17F, IL-22 and IL-23 receptor (53). Thus, RORγT inhibition seems to be an effective strategy in therapeutics of psoriasis. VTP-43742, an oral RORγT inhibitor is undergoing phase III study in treatment of plaque psoriasis. In a phase IIa study, 29 and 23% reduction in PASI was observed at 4 weeks in patients receiving 700 mg and 350 mg of VTP-43742 respectively along with 75% reduction in IL-17A and IL-17 F levels in both groups (54). Side effects included headache, flushing, elevated liver enzymes and nausea. Other agents such as JTE-451 and ABBV-157, oral RORγT inhibitors are currently in phase 2 and phase 1 of development respectively, for the treatment of moderate to severe psoriasis. New systemic and topical RORγT inhibitors may be the potential candidates for the treatment of psoriasis (55).

## Tyk2 Inhibitors

The TYK2, a JAK family gene, has been associated with psoriasis susceptibility genes and loss of function mutation is associated with various cytokine signaling defects that are implicated in psoriasis pathogenesis (56, 57). Individuals with these mutations have been found to be unaffected by immune-mediated inflammatory diseases without being susceptible to life-threatening infections (58). These observations suggested that TYK2 inhibitors may be a safe therapeutic target. BMS-986165 is an oral highly selective TYK2 inhibitor and inhibit STAT1 and STAT3 phosphorylation in peripheral blood mononuclear cells stimulated with IFN-α and IL-23 (6). BMS-986165 has shown good efficacy in psoriasis in phase II trials at doses 3 mg, 6 mg and 12 mg daily (59). Common adverse effects include headache, nausea, diarrhea, and upper respiratory tract infections (59). Phase III trials in plaque psoriasis and phase II trial in psoriatic arthritis are currently undergoing (6). Another selective TYK2 inhibitor, PF-06826647, is also being tested in moderate-to-severe psoriasis in an ongoing phase II clinical trial (NCT03895372) (6). Brepocitinib (formerly known as PF-06700841) is not a selective TYK2 inhibitor (rather a potent TYK2/JAK1 inhibitor), has shown good efficacy in phase II trials in psoriasis with few minor adverse effects, except thrombocytopenia and decreased reticulocyte count (60). A phase IIb study is currently undergoing to evaluate the efficacy and safety in psoriatic arthritis (6). A topical formulation is also being tested in mild to moderate psoriasis. These small molecules have advantages like oral route of administration, decreased cost, less immunologic adverse events as compared to biologics.

## Sphingtosine-1-Phosphate Receptor 1 (S1PR1) Antagonist

Sphingosine-1-phosphate (S1P) is involved in cell proliferation and survival, migration, inflammation and angiogenesis (61, 62). S1P inhibits the keratinocyte proliferations and increase cell differentiation (63). Ponesimod, an oral S1P receptor 1 antagonist leads to the downregulation of S1P receptor and prevent migration of lymphocytes from lymph nodes to skin in psoriasis (64). In a phase 2 study, PASI75 was achieved in 46 and 48% of patients receiving ponesimod 20 mg and 40 mg respectively as compared to placebo at 16-weeks and the improvement continued till 28 weeks (65). However, effect is not maintained after drug discontinuation due to its rapid elimination within 1 week. Adverse effects include transaminitis, shortness of breath, dizziness and may cause conduction abnormalities, thus contraindicated in patients with cardiac disease (65).

## A3 Adenosine Receptor Agonist

A3 adenosine receptors are G-protein coupled receptors involved in various intracellular pathways. These receptors have been found to be highly expressed on peripheral mononuclear cells in psoriasis patients (66). Piclidenoson, an oral A3 adenosine receptor agonist has been found to downregulate NF-κB signaling pathway and pro-inflammatory cytokines such as TNF-α, IL-6 and IL-12, and inhibit T-lymphocyte proliferation (67). In a phase II trial, a significant reduction in PASI was observed at 12 weeks as compared to placebo and drug was well tolerated (67). Currently, the drug is in phase III trials.

## mTOR Inhibitors

**T**he PI3-K/Akt/mTORC1 cascade acts as a regulator of epidermal homeostasis (68). Akt has been shown to be highly activated in skin of psoriatic lesions, except in the basal layer and mTOR, expression is found to be increased in lesional and Non-lesional skin of psoriasis patients (69, 70). An animal model study showed that the PUVA treatment led to improvement in psoriasis and normalization of mTORC1 signaling (71). This suggested a pathophysiological role of mTORC1 signaling in psoriasis. The increased expression of mTORC1 may have a role in increased proliferation of keratinocytes and decreased differentiation. During normal keratinization, mTORC1 signaling pathway is inactivated as the keratinocyte differentiation occurs (72). mTORC1 signaling also plays important roles in the innate and adaptive immunity (72, 73). Aberrant mTORC1 signaling was found in peripheral blood mononuclear cells (PBMCs) of psoriasis patients (74). Rapamycin, a mTOR inhibitor, has been used in few patients with psoriasis due to its antiproliferative and immunosuppressive actions (75). Everolimus was also used successfully in a psoriasis patient along with tacrolimus (76). Topical rapamycin has also been used in psoriasis showing clinical improvement (77). Thus, oral and topical mTOR inhibitors may be a successful therapeutic strategy in psoriasis and further research exploring the role of mTOR pathway as therapeutic target is warranted.

## Future Perspective

Recent advances in understanding the pathogenesis of the psoriasis has led to the development of newer therapies such as biologics and other small molecules. However, apert from the therapeutic options discussed, various other cells and pathways are implicated in the pathogenesis such as role of natural killer cells, regulatory T-cells and mesenchymal stem cells (MSCs). The regulatory T-cells have been found increased in lesional skin of psoriasis patients. Similarly, IL-10-producing regulatory B cells of psoriasis patients were reduced in number and showed decreased IL-10 production. MSCs have been implicated in the psoriasis pathogenesis and may serve as potential therapeutic target. MSCs have immunomodulatory properties and affect Th1 and Th17 lymphocytic inhibition in psoriatic skin (78). These MSCs have also been found to have pleiotropic effects of biologic therapy in psoriasis (79). MSCs based therapy has been tried in few patients with psoriasis with successful outcomes (80–82). However, larger studies are still needed to fully explore the role of these cells as a therapeutic option. Another class of drug i.e., selective serotonin reuptake inhibitors (SSRIs) have been found to be beneficial in psoriasis due to their anti-inflammatory properties and reduction in cytokine levels (83). Moreover, these agents prevent T-cell proliferation by reduced antigen presentation by dendritic cells and causes inflammatory cell apoptosis (83). Role of proanthocyanidins having antioxidant, anti-proliferative, antiangiogenic and anti-inflammatory properties as an therapeutic option needs to be investigated as oxidative stress plays an important role in the pathogenesis of psoriasis (84). A potent and selective NF-kB inducing kinase (NIK) inhibitor has been found effective in imiquimod induced psoriasis in animal model, highlighting the potential of newer strategy for the treatment of psoriasis (85). Mutations in CARD14 have been found in psoriasis patients (86). Such genetic associations indicate a role in immune regulatory pathways involved in psoriasis. Such observations may help in the better knowledge of psoriasis susceptibility genes and individualized approaches in management of psoriasis. In addition, the role of keratinocytes as initiators of psoriatic inflammation might further shift the focus to topical treatments. Further studies are needed to obtain better insights in the immunopathogenesis of the disease that may lead to the development of more targeted and more effective therapies.

## Conclusion

Many novel systemic and topical therapies are currently in development. The success of these agents depends on the efficacy and safety of these drugs in future studies. Better understanding of inflammatory pathways involved the pathogenesis and newer discoveries may lead to the effective therapeutic strategies in management of psoriasis.

## Author Contributions

VT: study design, acquisition, analysis or interpretation of data, and drafting of the manuscript. RM: study concept and design, acquisition, analysis or interpretation of data, drafting of the manuscript, critical revision of the manuscript for important intellectual content, statistical analysis, administrative, technical, or material support, and study supervision. All authors contributed to the article and approved the submitted version.

## Conflict of Interest

The authors declare that the research was conducted in the absence of any commercial or financial relationships that could be construed as a potential conflict of interest.

## Publisher's Note

All claims expressed in this article are solely those of the authors and do not necessarily represent those of their affiliated organizations, or those of the publisher, the editors and the reviewers. Any product that may be evaluated in this article, or claim that may be made by its manufacturer, is not guaranteed or endorsed by the publisher.
